# Effects of Foliar Redox Status on Leaf Vascular Organization Suggest Avenues for Cooptimization of Photosynthesis and Heat Tolerance

**DOI:** 10.3390/ijms19092507

**Published:** 2018-08-24

**Authors:** Jared J. Stewart, Christopher R. Baker, Carlie S. Sharpes, Shannon Toy Wong-Michalak, Stephanie K. Polutchko, William W. Adams, Barbara Demmig-Adams

**Affiliations:** 1 Department of Ecology & Evolutionary Biology, University of Colorado, Boulder, CO 80309-0334, USA; carlie.sharpes@colorado.edu (C.S.S.); stephanie.polutchko@colorado.edu (S.K.P.); william.adams@colorado.edu (W.W.A.); barbara.demmig-adams@colorado.edu (B.D.-A.); 2 School of Education, University of Colorado, Boulder, CO 80309-0249, USA; 3 Department of Plant & Microbial Biology, University of California, Berkeley, CA 94720-3102, USA; cbaker@berkeley.edu (C.R.B.); swongmichalak@berkeley.edu (S.T.W.-M.)

**Keywords:** *Arabidopsis*, antioxidant, C-repeat binding factor, phloem, photoprotection, photosynthesis, PsbS, tocopherol, xylem, zeaxanthin

## Abstract

The interaction of heat stress with internal signaling networks was investigated through *Arabidopsis* *thaliana* mutants that were deficient in either tocopherols (*vte1* mutant) or non-photochemical fluorescence quenching (NPQ; *npq1*, *npq4*, and *npq1 npq4* mutants). Leaves of both *vte1* and *npq1 npq4* mutants that developed at a high temperature exhibited a significantly different leaf vascular organization compared to wild-type Col-0. Both mutants had significantly smaller water conduits (tracheary elements) of the xylem, but the total apparent foliar water-transport capacity and intrinsic photosynthetic capacity were similarly high in mutants and wild-type Col-0. This was accomplished through a combination of more numerous (albeit narrower) water conduits per vein, and a significantly greater vein density in both mutants relative to wild-type Col-0. The similarity of the phenotypes of tocopherol-deficient and NPQ-deficient mutants suggests that leaf vasculature organization is modulated by the foliar redox state. These results are evaluated in the context of interactions between redox-signaling pathways and other key regulators of plant acclimation to growth temperature, such as the C-repeat binding factor (CBF) transcription factors, several of which were upregulated in the antioxidant-deficient mutants. Possibilities for the future manipulation of the interaction between CBF and redox-signaling networks for the purpose of cooptimizing plant productivity and plant tolerance to extreme temperatures are discussed.

## 1. Introduction

Today’s changing climate threatens crop productivity through unpredictable weather events and warmer, drier summers in many regions [1]. Photosynthesis, as the engine of plant productivity, depends on a constant water supply to replace water that is lost from the leaves during CO_2_ uptake [2,3]. Thus, the maintenance of photosynthetic productivity requires a vascular system with a sufficient capacity for water transport and resistance to the introduction of air bubbles (embolisms) when evaporative demand exceeds water supply [4] or during freeze–thaw cycles [5]. Much of the insight into the link between photosynthesis and long-distance water transport has come from studies of woody species [4,5]. We have recently focused on leaves of herbaceous species, for which we reported concomitant adjustments in leaf vascular anatomy and photosynthetic capacity in response to growth temperature (see, e.g., [6]). For example, leaves of *Arabidopsis thaliana* that were grown under hot versus cool temperature exhibited a foliar vascular network with more numerous veins and an increased proportion of water conduits relative to sugar conduits [7,8]. There is also evidence of genetic differences among herbaceous species that are active during different times of the year. Summer annuals that germinate in the spring and grow over the summer exhibited a foliar vasculature with constitutively more numerous veins and a greater ratio of water-to-sugar conduits compared to winter annuals that germinate in the fall, overwinter, and set seed in the spring before being subjected to the heat of summer [8,9].

Plant growth and stress tolerance is orchestrated by gene regulators, including phytohormones and transcription factors, many of which receive input from signaling networks that sense the state of the environment [1,10,11]. Environmental cues are sensed by multiple redox pathways—which generate oxidant-based and antioxidant-based signals—interacting with gene regulators [12]. One example for a transcription factor family that orchestrates adjustments in plant form and function in response to growth temperature and water availability is C-repeat binding factors (CBFs, also referred to as dehydration-responsive element-binding 1 (DREB1) transcription factors [13,14]). The CBFs and other DREB transcription factors closely interact with phytohormones and redox-signaling networks. While reactive oxygen species (ROS) have traditionally been regarded as generally harmful and antioxidants as generally protective, this view is now challenged in reviews such as one entitled simply “ROS are good” [15]. This revised view focuses on the essential roles of ROS-based signals in communicating fluctuations in the environment as well as coordinating plant response to these fluctuations (see, e.g., [16,17]).

We reported that the *A. thaliana* mutant *vte1* deficient in tocopherols exhibited more numerous foliar minor veins with a greater proportion of water conduits compared to wild-type Col-0 when exposed to intermittently elevated temperatures during growth [18]. Tocopherols are well-known antioxidants (see [19]), but they have also been suggested to act through additional, redox-independent signaling pathways [20]. We here address several follow-up questions. Do other mutant systems that are deficient in antioxidant processes other than tocopherols exhibit similar effects on the leaf vasculature as the tocopherol-deficient *vte1* mutant when grown under hot temperature? Is there thus evidence that adjustments in leaf vascular organization are linked to redox-dependent signaling? If mutants deficient in antioxidant processes other than the tocopherol-based system also exhibit a different foliar vascular organization compared to wild-type Col-0, are the specific vascular features the same in *vte1* and other antioxidant-deficient mutants? Does antioxidant deficiency impact *CBF* expression? How do the foliar vascular features of antioxidant-deficient *A. thaliana* mutants compare to species adapted to hot/dry environments in the context of water-transport capacity versus cavitation risk?

In the present study, we characterized the foliar vasculature of the *vte1* mutant as well as three *A. thaliana* mutants that were deficient in thermal dissipation in the chloroplast (assessed from non-photochemical quenching of chlorophyll fluorescence, NPQ). The three *npq* mutants were *npq1*, *npq4*, and the double mutant *npq1 npq4*, which were all grown under a high-temperature regime of 35 °C. The role of photoprotective thermal dissipation is the preemptive removal of surplus excitation energy before this energy can be transferred from chlorophyll to oxygen, forming the ROS singlet oxygen [21], and to the lowered production of other redox signals [22,23]. Since thermal energy dissipation requires the xanthophyll pigment zeaxanthin and the PsbS (photosystem II subunit S) protein, NPQ is lower in the *A. thaliana* mutant *npq1*, as it is missing the violaxanthin de-epoxidase that converts the precursor violaxanthin to zeaxanthin [24], and also in the *A. thaliana* mutant *npq4,* missing the PsbS protein [25]. The antioxidant actions of thermal dissipation and tocopherols can intersect in several ways (for a summary of the literature supporting the following actions, see [23]). While thermal energy dissipation removes excess excitation energy, tocopherols detoxify the singlet oxygen that is formed by the energy transfer from chlorophyll to oxygen. During changes in the environment that cause leaves to absorb light levels exceeding the combined capacities of photochemistry, zeaxanthin-associated thermal energy dissipation (quantified as NPQ), and tocopherol-supported ROS detoxification, the level of ROS increase and trigger acclimatory responses via multiple redox-signaling pathways. Dogra et al. [26] provided a review of several redox-signaling pathways linked to singlet oxygen. One of these signaling pathways involves the peroxidation of polyunsaturated membrane lipids by singlet oxygen to several gene regulators. In the absence of either thermal energy dissipation or tocopherols, greater levels of these gene regulators are formed. In addition to being involved in thermal energy dissipation and the detoxification of ROS, tocopherols and zeaxanthin also cooperate in re-reducing oxidized membrane lipids, and thereby further lower the production of lipid peroxidation-based signals.

## 2. Results

### 2.1. Non-photochemical Quenching and Foliar Pigment Levels in Mutants Deficient in Thermal Energy Dissipation or in Tocopherols

**Figure 1:**
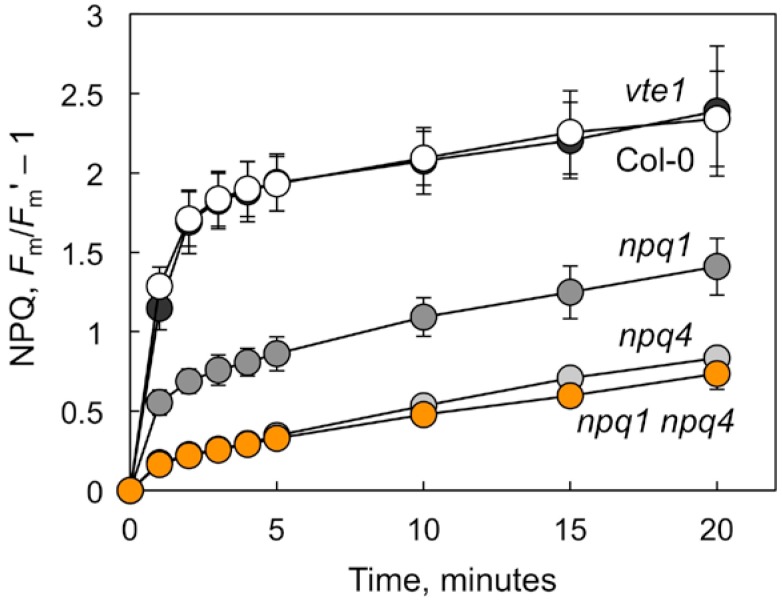
Time course of the development of non-photochemical quenching of chlorophyll fluorescence (NPQ) under 2% O_2_ (balance N_2_) and 2000 µmol photons m^−2^ s^−1^ at 25 °C. Mean values ± standard deviation, *n* = 3 or 4, from the leaves of wild-type Col-0 (open symbols) and *vte1* (dark gray symbols), *npq1* (medium gray symbols), *npq4* (light gray symbols), and *npq1 npq4* (orange symbols) mutants of *Arabidopsis thaliana* grown at 35 °C. Differences between Col-0 and the *npq1*, *npq4*, and *npq1 npq4* mutants at all of the time points were highly significant (*p* < 0.001; Student’s *t*-test). There were no significant differences between Col-0 and *vte1*.

Figure 1 illustrates the degree of deficiency in thermal energy dissipation, as quantified from non-photochemical fluorescence quenching (NPQ) in the absence of CO_2_ and with just enough oxygen (2%) to support intrathylakoid acidification (thereby minimizing photosynthetic electron transport while maximizing energy dissipation), in several NPQ-deficient mutants. While *npq1*—which is unable to produce zeaxanthin from its precursor violaxanthin (Table 1)—exhibited significantly reduced NPQ compared to the wild-type Col-0; an even more pronounced decrease in NPQ was seen in the *npq4* that lacked the PsbS protein (Figure 1). In the double mutant *npq1 npq4*, zeaxanthin formation (Table 1) and NPQ (Figure 1) were inhibited to the same extent as in *npq4*. In contrast to the *npq* mutants, the tocopherol-deficient *vte1* mutant plants exhibited similar foliar carotenoid levels (Table 1) and energy dissipation capacity (Figure 1) as the Col-0 genotype. Two other foliar carotenoids with additional roles in photoprotection and antioxidation [27,28], β-carotene and lutein, were present at similar levels in Col-0 and the *vte1* and *npq* mutants (Table 1).

### 2.2. Impact of Foliar Antioxidant Status on Leaf Vascular Organization

**Figure 2:**
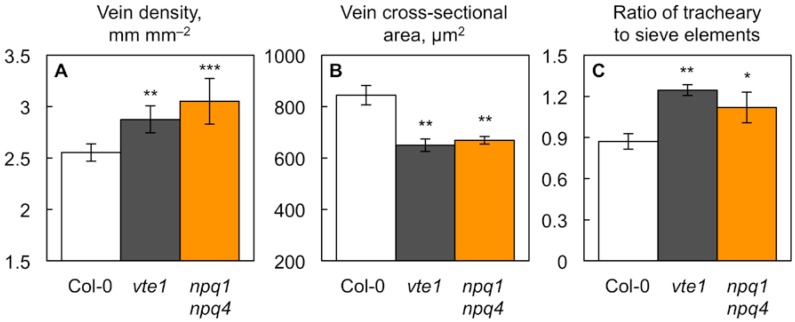
(**A**) Minor vein density (vein length per leaf area), (**B**) minor vein cross-sectional area, and (**C**) the ratio of the number of water-transporting tracheary to sugar-exporting sieve elements in the leaves of wild-type Col-0 (open columns) and the *vte1* (dark gray columns) and *npq1 npq4* (orange columns) mutants of *Arabidopsis thaliana* grown at 35 °C. Mean values ± standard deviations for (**A**) and ± standard errors for (**B**,**C**) (*n* = 3 or 4). Significant differences (Student’s *t*-test) between Col-0 and the mutants are indicated by asterisks; * = *p <* 0.05, ** = *p <* 0.01, *** = *p <* 0.001.

**Figure 3:**
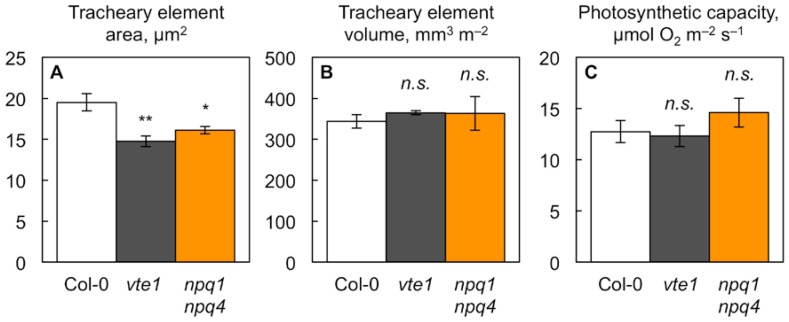
(**A**) Individual tracheary element cross-sectional area, (**B**) total tracheary element volume per leaf area, and (**C**) photosynthetic capacity (light-saturated and CO_2_-saturated rate of oxygen evolution determined at 25 °C) from leaves of wild-type Col-0 (open columns) and the *vte1* (dark gray columns) and *npq1 npq4* (orange columns) mutants of *Arabidopsis thaliana* grown at 35 °C. Mean values ± standard errors for (**A**,**B**) and ± standard deviations for (**C**) (*n* = 3 or 4). Significant differences (Student’s *t*-test) between Col-0 and the mutants are indicated by asterisks; * = *p <* 0.05, ** = *p <* 0.01, *n.s.* = not significantly different.

Neither of the single *npq* mutants (*npq1* or *npq4*) exhibited significantly different foliar vein densities (mean values ± standard deviations of 2.58 ± 0.14 (*n* = 4) and 2.67 ± 0.09 (*n* = 3) mm mm^−2^ for *npq1* and *npq4*, respectively) compared to wild-type Col-0 (see Figure 2A). On the other hand, both the double mutant *npq1 npq4* and the tocopherol-deficient *vte1* mutant did exhibit significantly altered minor vein features compared to wild-type Col-0 (Figure 2 and Figure 3, Table 2). Both of the latter mutants had more numerous (Figure 2A) but smaller minor veins (with significantly smaller cross-sectional areas; Figure 2B) compared to wild-type Col-0. Furthermore, the ratio of water conduits (tracheary elements) to sugar conduits (sieve elements) was greater in the minor veins of the *vte1* and *npq1 npq4* mutants compared to wild-type Col-0 (Figure 2C).

A closer examination revealed that individual water conduits also had smaller cross-sectional areas in both mutants compared to wild-type Col-0 (Figure 3A). Figure 3B shows that, despite exhibiting narrower water conduits, both mutants had the same total water conduit volume per leaf area as wild-type Col-0. The total water conduit volume per leaf area is a proxy for the leaf’s capacity to transport water, and can be obtained by multiplying total cross-sectional water conduit area per minor vein × vein density. In addition, photosynthetic capacity was similarly high in the two mutants compared to wild-type Col-0 (Figure 3C). How is it possible that the two mutants have narrower individual water conduits, and yet the same total water-conduit volume, and apparent water transport capacity, per leaf area as wild-type Col-0? The explanation lies in both mutants compensating for their narrower water conduits with greater numbers of water conduits on a leaf area basis. Table 2 shows that the smaller minor veins in both mutants had fewer sugar conduits per vein, but a similar or slightly greater number of water conduits per vein. The normalization for vein density (multiplying conduit number per vein × vein density) shows that the water conduit number normalized for vein density was significantly greater in both mutants, while the sugar conduit number normalized for vein density was the same in both mutants compared to wild-type Col-0 (Table 2).

### 2.3. Comparison of Antioxidant-Deficient Mutants with a Pair of Natural A. thaliana Accessions Differing in NPQ and Tocopherol Levels

**Figure 4:**
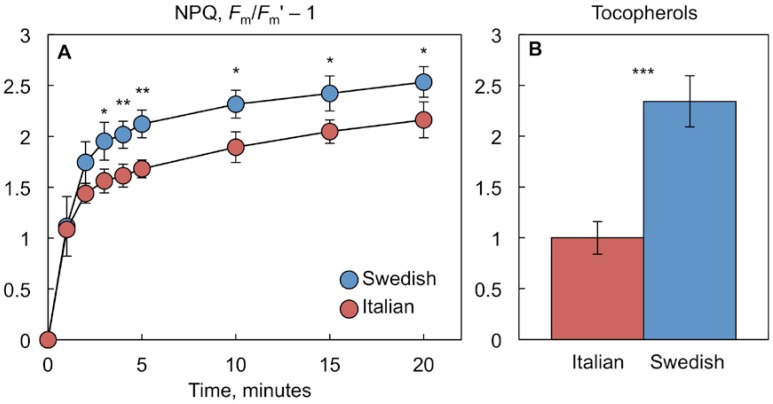
(**A**) Time course of the development of the non-photochemical quenching of chlorophyll fluorescence (NPQ) under 2% O_2_ (balance N_2_) and 2000 µmol photons m^−2^ s^−1^ at 25 °C and (**B**) tocopherol content for leaves of Italian (red symbols) and Swedish (blue symbols) ecotypes of *Arabidopsis thaliana* grown at 35 °C. Tocopherols are recalculated from data in Stewart et al. [8] and expressed as relative values (with the mean value for the Italian ecotype set to 1). Mean values ± standard deviations (*n* = 3 or 4). Significant differences (Student’s *t*-test) between the ecotypes are indicated by asterisks; * = *p <* 0.05, ** = *p <* 0.01, *** = *p <* 0.001.

We further compared the *vte1* and *npq1 npq4* mutants to a pair of natural *A. thaliana* accessions from Sweden and Italy that differed in NPQ [29,30], tocopherol levels [8], and in a member of the CBF transcription factor family ([31]; see also below). Figure 4A shows that hot-grown plants of the Swedish ecotype exhibited a significantly greater NPQ capacity than the Italian ecotype. Figure 4B presents data replotted from Stewart et al. [8] demonstrating greater tocopherol levels in hot-grown plants of the Swedish compared to the Italian ecotype.

**Figure 5:**
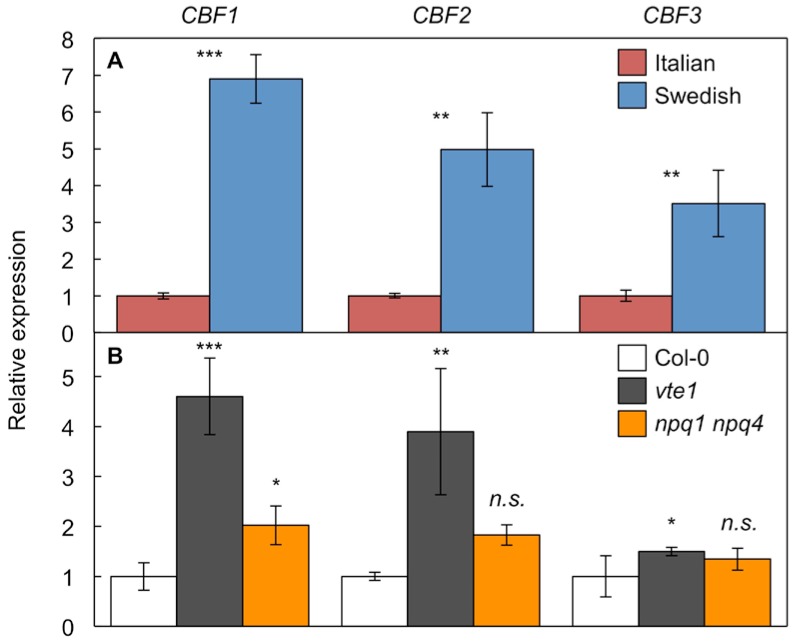
Relative expression of *CBF1*, *CBF2*, and *CBF3* genes from the leaves of (**A**) Italian (red columns; mean transcript level set to 1) and Swedish (blue columns) ecotypes and (**B**) wild-type Col-0 (open columns; mean transcript levels set to 1) and the *vte1* (dark gray columns) and *npq1 npq4* (orange columns) mutants of *Arabidopsis thaliana* grown at 35 °C. Mean values ± standard deviations (*n* = 3 or 4); significant differences (Student’s *t*-test) between (**A**) ecotypes or (**B**) mutants and wild-type, respectively, indicated by asterisks; * = *p <* 0.05, ** = *p <* 0.01, *** = *p <* 0.001, *n.s.* = not significantly different.

The Italian ecotype carries a *CBF2* mutation that renders the CBF2 transcription factor non-functional, which results in lower freezing tolerance of the Italian ecotype [31]. Figure 5 shows greater expression levels of *CBF1*, *CBF2*, and *CBF3* in hot-grown plants of not only the Swedish compared to the Italian ecotype (Figure 5A), but also of the antioxidant-deficient *vte1* and *npq1 npq4* mutants compared to the wild-type Col-0 (Figure 5B). These results suggest that distinct leaf antioxidant and vascular phenotypes can be associated with alterations in *CBF* expression. Greater *CBF* expression was associated with greater antioxidant levels and relatively similar vascular anatomy [8] in the Swedish ecotype relative to the Italian ecotype. This ecotypic difference is contrasted with the pairing of greater *CBF* expression with lower antioxidant levels and more numerous, smaller veins and water conduits in *vte1* and *npq1 npq4* relative to Col-0.

## 3. Discussion

### 3.1. Vascular Phenotype in Antioxidant Mutants

Mutants deficient in two distinct antioxidant processes, tocopherols (*vte1* mutant) and photoprotective thermal energy dissipation (*npq1 npq4* mutant), exhibited a similar phenotype with respect to foliar vascular organization in plants grown under hot temperature: smaller, yet more numerous minor veins with narrower, yet more numerous water conduits. The water-conduit diameter is associated with the risk for embolisms, especially under exposure to freeze–thaw cycles [5,32,33]. When water freezes, gas is forced out of solution; in narrow water conduits, the resulting gas bubbles are smaller and more likely to be redissolved upon thawing [34]. Under high negative pressure in the xylem, air can be pulled into water conduits through porous cell-wall sections [35]. Embolism protection under high evaporative demand has been shown to depend on features of the junctions between neighboring vessels that control the movement of air from embolized to water-filled conduits ([36]; see also [37]). Overall, multiple features contribute to hydraulic safety. An additional feature is the ratio of water-conduit wall thickness to the lumen area [35,38,39,40], and several studies (e.g., [40,41]) have invoked links between narrower foliar water conduits and drought tolerance.

It is attractive to speculate that both of the antioxidant-deficient mutants may have a lower risk for embolism/cavitation under freeze–thaw cycles and/or drought. Future studies should assess the additional anatomical features of xylem conduits in the leaves and stems of these systems as well as their hydraulic function. The greater number of water conduits in both *vte1* and *npq1 npq4*, which is sufficient to result in unaltered apparent water-transport capacity compared to the wild-type Col-0, could serve to combine the potential advantages of narrower water conduits with the maintenance of high capacities for both water transport and photosynthesis.

In addition to the greater water-conduit numbers per vein, the significantly greater vein density in both mutants compared to wild-type Col-0 helps to compensate for the mutants’ smaller water conduit size. High vein density is often associated with enhanced drought tolerance. For example, Dunbar-Co et al. [42] showed that the species of the genus *Plantago* originating from drier sites had higher vein densities compared to congeneric species from moister sites, and a similar trend was seen for *A*. *thaliana* ecotypes originating from sites of differing precipitation and grown under controlled conditions [7,10].

The similarity of the foliar vascular phenotype of *vte1* and the *npq1 npq4* double mutant demonstrated here suggests that this phenotype is produced by a common underlying redox-regulation network rather than a regulatory pathway unique to tocopherol-based signaling. What redox-related processes are affected by both tocopherols and thermal energy dissipation? Deficiency in both tocopherols and energy dissipation impact the production of reactive oxygen species, in particular singlet oxygen [19,21]. Increased production of singlet oxygen, in turn, can lead to the increased production of messengers such as the plant hormone jasmonic acid, which is formed from products of enzymatic lipid peroxidation, even when there is negligible non-enzymatic lipid peroxidation (for a summary, see [23]).

Why is foliar vascular organization in the *npq4* single mutant the same as in wild-type Col-0, but significantly different between wild-type Col-0 and the *npq1 npq4* double mutant (Figure 2 and Figure 3), even though there is no difference in NPQ capacity between these two *npq* mutant lines (Figure 1)? The greater impact on leaf vasculature of *npq1 npq4* compared to *npq4* alone could involve the non-NPQ effects of zeaxanthin [43]. The single *npq4* mutant differs from the double mutant *npq1 npq4* in its greater zeaxanthin content (Table 1). Zeaxanthin can serve as a direct quencher of singlet oxygen, and acts synergistically with tocopherols in the rereduction of lipid radicals (see [22,43,44]).

### 3.2. Interaction of Redox Signaling Networks with other Regulators

In the pairing of hot-grown plants of the Swedish versus Italian ecotypes of *A. thaliana* that did not differ significantly in their foliar vascular organization [8], lower antioxidant levels were associated with lower *CBF* expression in the Italian ecotype. In contrast, in the pairing of hot-grown Col-0 versus *vte1* and *npq1 npq4* mutants with their more numerous, smaller veins and water conduits, lower antioxidant levels were associated with greater *CBF* expression in the mutants. Taken together, these findings suggest that foliar redox state, rather than *CBF* expression level *per se*, is a major regulator of leaf vascular organization in plants grown under a hot-temperature regime. Specifically, the altered foliar vascular organization of *vte1* and *npq1 npq4* appears to depend chiefly on these mutants’ low antioxidant status. The adjustment of multiple aspects of plant phenotype in response to the temperature environment is orchestrated by interaction among gene regulators such as the CBFs, redox-signaling networks, and phytohormones [1,13,45,46,47]. The finding of enhanced *CBF1–3* expression in the antioxidant-deficient *vte1* and *npq1 npq4* mutants is consistent with a stimulation of *CBF1–3* expression by oxidant-derived messengers. Likewise, the finding of enhanced photoprotection and antioxidant levels in the Swedish ecotype (which expresses *CBF1–3* more highly than the Italian ecotype) is consistent with the regulation of antioxidant pathways by CBF transcription factors.

The present findings support the notion that ROS play critical roles in plant development and stress tolerance (see [12,48]). In particular, the current study supports the role of oxidant-based signaling in heat tolerance via the maintenance of a high foliar capacity for water movement with potentially enhanced cavitation resistance. It may be rewarding for future efforts to improve crop heat and drought tolerance through breeding and gene editing to aim for combining the independent manipulation of redox-signaling networks with the direct manipulation of DREBs or other transcription factors that orchestrate plant temperature and drought tolerance, such as stress-responsive no apical meristem (NAM), Arabidopsis transcription activation factor (ATAF), and cup-shaped cotyledon (CUC) (collectively known as NAC), homeodomain-steroidogenic acute regulatory protein-related lipid transfer (HD-START), nuclear factor-Y (NF-Y), and HARDY (for more information and examples, see [49]). This could be done, for example, by targeting both the CBF-binding promoter regions of antioxidant genes and the redox messenger-binding promoter regions of redox-modulated transcription factors such as CBFs and other DREBs.

### 3.3. Comparison of Antioxidant-Deficient Phenotype with Phenotype of Species Adapted to Specific Environments

Both the Swedish and Italian ecotypes of *A. thaliana* grow as winter annuals, and neither ecotype experiences extremely hot temperatures in their respective habitats of geographic origin [7]. The Italian ecotype flowers early in the spring and completes its life cycle before the summer heat sets in. Enhanced cavitation resistance is important for both cold tolerance [50] and heat/drought tolerance [39,40]. It is noteworthy that a biennial weed that grows vigorously throughout all seasons, *Malva neglecta* [51], exhibits a leaf vascular phenotype [52] reminiscent of that of the *vte1* and *npq1 npq4* mutants shown here. *Malva neglecta* features exceptionally numerous veins with numerous narrow water conduits [52], which may contribute to its productivity throughout periods of intense heat and cold alike. It may be rewarding to look for unique adaptations in redox-signaling and related networks in highly specialized plant systems, such as biennials that combine superior photosynthetic productivity under both cold and heat stress.

## 4. Materials and Methods 

### 4.1. Plant Material and Growth Conditions

Wild-type Columbia-0 (Col-0) *Arabidopsis thaliana* was compared with the mutant lines of the same genetic background deficient in tocopherols (*vte1-1* [53]), violaxanthin deepoxidase (*npq1-2* [54]), or photosystem II subunit S (*npq4-1* [25]), as well as a mutant line deficient in both of the latter two (*npq1-2 npq4-1* [43]). All of the plants were grown from seed in Conviron E15 growth chambers (Controlled Environments Ltd., Winnipeg, MB, Canada) under a 9-h photoperiod of 250–300 µmol photons m^−2^ s^−1^. Seeds were vernalized at 4 °C in H_2_O for four days and germinated in six-pack seed starting trays containing 50 mL of soil (Fafard Growing Mix 2; Sun Gro Horticulture, Agawam, MA, USA), after which individual seedlings were transplanted with soil into larger (2.9-L) individual pots. Air temperatures were set at 25 °C during the photoperiod and 20 °C during the dark period while the seeds germinated, and were increased to 35 °C during the photoperiod (resulting in a leaf temperature of 35.6 ± 0.1 °C (mean value ± standard error; *n* = 15)), and 25 °C during the dark period two days after the seedlings had been transplanted. Relative humidity during the photoperiod was 49.9 ± 0.5% (mean value ± standard deviation (*n* = 36); this was measured at 5-min intervals over the middle 3 h of the photoperiod before the harvest), resulting in an estimated vapor pressure deficit of 2.97 ± 0.15 kPa (mean value ± standard deviation; *n* = 15). Plants were watered daily and received nutrients every other day, as previously described [55].

For the selected parameters, *Arabidopsis thaliana* ecotypes from Italy (Castelnuovo-12 (ABRC stock number: CS98761), sub-line 24) and Sweden (Rodasen-47 (ABRC stock number: CS98762), sub-line 29) were also compared. These plants had been grown for a prior separate investigation [8] at 35 °C (light)/25 °C (dark) under a 9-h photoperiod of 400 µmol photons m^−2^ s^−1^. For more details regarding these prior experiments, see [8]. For more details on these ecotypes, see [7,56].

### 4.2. Photosynthesis and Chlorophyll Fluorescence

Photosynthetic capacity was determined as light-saturated and CO_2_-saturated oxygen evolution under 2000 µmol photons m^−2^ s^−1^ and a water-saturated atmosphere containing 5% CO_2_ (21% O_2_, balance N_2_; which bypasses all resistance to CO_2_) in leaf disc oxygen electrodes (Hansatech Instruments Ltd., King’s Lynn, Norfolk, UK; [57]) coupled to circulating water baths (Fisher Scientific, Hampton, NJ, USA) set to 25 °C. Non-photochemical quenching of chlorophyll fluorescence (NPQ) was determined using a PAM-101 chlorophyll fluorometer (Walz, Effeltrich, Germany), modified Hansatech leaf disc oxygen electrodes, circulating water baths set to 25 °C, and a gas stream without CO_2_ (2% O_2_, balance N_2_; which limits linear electron transport but allows build-up of the trans-thylakoid pH gradient for NPQ; for further background on this approach, see [58]). Maximal fluorescence yields were elicited via saturating light pulses in leaves darkened for 5 min (*F*_m_) and again during illumination (*F*_m_‘) with 2000 µmol photons m^−2^ s^−1^ for 1 min, 2 min, 3 min, 4 min, 5 min, 10 min, 15 min, and 20 min. NPQ was calculated using the equation *F*_m_/*F*_m_‘ − 1 (see [59]).

### 4.3. Pigments

Chlorophylls and carotenoids were determined via high-performance liquid chromatography as previously described [29] from leaf discs (0.30 cm^2^) that were collected immediately following measurements of NPQ, as described above.

### 4.4. Minor Vein Anatomy

Vein density was quantified as minor vein length per unit of leaf area from leaf segments chemically cleared with 70% (*v*/*v*) ethanol followed by 5% (*w*/*v*) NaOH. Vascular cell numbers and cross-sectional areas were determined as previously described [60] from leaf segments (approximately 2 mm × 2 mm) fixed with glutaraldehyde and paraformaldehyde, which were dehydrated through an acetone series, and embedded in Spurr resin [61], as previously described [62]. All of the anatomical measurements (e.g., cross-sectional area) were made with ImageJ [63] from images produced with an Axioskop 20 light microscope (Carl Zeiss AG, Oberkochen, Germany) and an OptixCam Summit Series digital camera (The Microscope Store, LLC, Roanoke, VA, USA).

### 4.5. Gene Expression

Two leaf discs (0.73 cm^2^) from the same plant, which were taken immediately before the onset of the photoperiod, were homogenized in liquid nitrogen by bead beating, and RNA was extracted and DNase-treated (RNeasy Plant Mini Kit; Qiagen, Hilden, Germany). cDNA synthesis was performed with 2 µg of DNase-treated RNA per sample (Omniscript cDNA synthesis kit; Qiagen, Hilden, Germany). Due to the sequence similarity of the three *CBF* genes, qPCR primers were designed using the NCBI Primer-BLAST Tool to target the 3′ UTR region for each gene in order to minimize the off-target amplification of paralogous genes. qPCR was performed with 40 ng of cDNA per sample (Fast Sybr Green Master Mix; Applied Biosystems, Foster City, CA, USA), and the housekeeping gene *UBC21* (*AT5G25760*) was used as a control gene.

### 4.6. Statistical Analysis

Comparison of the mean values between each mutant line and wild-type Col-0 and between the Swedish and Italian ecotypes were made via Student’s *t*-tests with JMP Pro 14.0.0 statistical software (SAS Institute Inc., Cary, NC, USA).

## Figures and Tables

**Table 1:** Foliar zeaxanthin, lutein, and β-carotene levels immediately following the NPQ measurements shown in Figure 1 for wild-type Col-0 and the *vte1*, *npq1*, *npq4*, and *npq1 npq4* mutants of *Arabidopsis thaliana* grown at 35 °C.

Carotenoid Levels	Col-0	*vte1*	*npq1*	*npq4*	*npq1 npq4*
Zeaxanthin, mmol mol^−1^ Chl	35 ± 1	35 ± 1	2.1 ± 0.5 ***	35 ± 3	2.1 ± 0.3 ***
Zeaxanthin, % of VAZ pool	65 ± 1	64 ± 2	3.9 ± 0.9 ***	63 ± 3	4.0 ± 0.7 ***
Lutein, mmol mol^−1^ Chl	142 ± 1	145 ± 4	142 ± 0	142 ± 2	142 ± 0
β-carotene, mmol mol^−1^ Chl	83 ± 5	82 ± 3	87 ± 2	86 ± 2	83 ± 5

Mean values ± standard deviations (*n* = 3). Significant differences (Student’s *t*-test) between mutants and wild-type Col-0 are indicated by asterisks; *** = *p* < 0.001. Chl = chlorophyll *a* + *b*, VAZ = Violaxanthin + Antheraxanthin + Zeaxanthin.

**Table 2:** Number of water-transporting tracheary elements and sugar-exporting sieve elements (per minor vein and also normalized for vein density; from Figure 2A) in the leaves of wild-type Col-0 and the *vte1* and *npq1 npq4* mutants of *Arabidopsis thaliana* grown at 35 °C.

Vascular Features	Col-0	*vte1*	*npq1 npq4*
Tracheary elements per minor vein	6.8 ± 0.4	8.6 ± 0.5 *	7.2 ± 0.5
Tracheary elements per minor vein × vein density	17 ± 1	25 ± 1 **	22 ± 1 *
Sieve elements per minor vein	7.8 ± 0.0	7.0 ± 0.1 *	6.6 ± 0.4 **
Sieve elements per minor vein × vein density	20 ± 0	20 ± 0	20 ± 1

Mean values ± standard errors (*n* = 3 or 4). Significant differences (Student’s *t*-test) between mutants and wild-type Col-0 are indicated by asterisks; * = *p* < 0.05, ** = *p* < 0.01.

## Abbreviations

ABRC	Arabidopsis Biological Resource Center
cDNA	Complementary deoxyribonucleic acid
CBF	C-repeat binding factor
Col-0	Columbia-0 (a wild-type *Arabidopsis thaliana* line)
DREB	Dehydration-responsive element binding factor
*F*_m_	Maximal chlorophyll fluorescence determined in leaves darkened for 5 min
*F*_m_‘	Maximal chlorophyll fluorescence determined in a leaf exposed to light
MDPI	Multidisciplinary Digital Publishing Institute
NCBI	National Center for Biotechnology Information
NPQ	Non-photochemical quenching of chlorophyll fluorescence calculated as *F*_m_/*F*_m_‘ − 1
*npq1*	An *Arabidopsis thaliana* mutant line deficient in violaxanthin deepoxidase
*npq1 npq4*	An *Arabidopsis thaliana* mutant line deficient in both violaxanthin deepoxidase and PsbS
*npq4*	An *Arabidopsis thaliana* mutant line deficient in the PsbS protein
*n.s.*	Not significantly different
PsbS	Photosystem II subunit S
qPCR	Quantitative polymerase chain reaction
RNA	Ribonucleic acid
ROS	Reactive oxygen species
*UBC21*	Gene for ubiquitin-conjugating enzyme E2 21
UTR	Untranslated region
VAZ	Violaxanthin + Antheraxanthin + Zeaxanthin
*vte1*	An *Arabidopsis thaliana* mutant line deficient in tocopherols
